# Green synthesis of silver nanoparticles using neem and turmeric extract and its antimicrobial activity of plant mediated silver nanoparticles

**DOI:** 10.1016/j.jobcr.2025.02.005

**Published:** 2025-02-17

**Authors:** Rohit Kumar Singh, Deepak Nallaswamy, Shanmugam Rajeshkumar, Sheeja S. Varghese

**Affiliations:** a Saveetha Dental College and Hospitals, Saveetha Institute of Medical and Technical Sciences, Chennai, 600077, TN, India; b Nanobiomedicine Lab, Department of Anatomy, Saveetha Medical College and Hospital, Saveetha Institute of Medical and Technical Sciences, Chennai, 600077, TN, India

**Keywords:** Green synthesis, Silver nanoparticles, Eco-friendly, Antimicrobial agent

## Abstract

**Introduction:**

The green synthesis of silver nanoparticles has gained attention for being environmentally friendly and cost-effective. This study investigates the synthesis of silver nanoparticles using neem and turmeric extracts, which serve as natural reducing and capping agents, with a focus on characterizing these nanoparticles and assessing their antimicrobial properties against oral pathogens.

**Materials and methods:**

Neem and turmeric extracts were prepared by heating their powdered forms in distilled water, followed by filtration. The extracts were then mixed with a silver nitrate solution, and the reaction was stirred for 24–48 h. The resulting nanoparticles were characterized using UV–Visible spectroscopy, SEM, EDAX, and XRD analysis. The antimicrobial activity of the nanoparticles was tested against four oral pathogens using the agar well diffusion method.

**Results:**

Successful synthesis of silver nanoparticles was confirmed by a color change and characterization analyses. UV–Visible spectroscopy showed a peak at 440 nm, indicating nanoparticle formation. SEM revealed spherical and uniform nanoparticles, while EDAX confirmed the presence of silver. XRD analysis showed the crystalline nature of the nanoparticles, with sizes ranging from 4 nm to 14.81 nm. The nanoparticles exhibited significant antimicrobial activity against *Staphylococcus aureus*, Streptococcus mutans, and Lactobacillus species, but were less effective against Candida albicans.

**Conclusion:**

The study demonstrates the effectiveness of neem and turmeric extracts in the green synthesis of silver nanoparticles, which exhibited notable antimicrobial activity. This research underscores the potential of plant-mediated synthesis for developing eco-friendly antimicrobial agents.

## 1 Introduction

**Graph 1:**
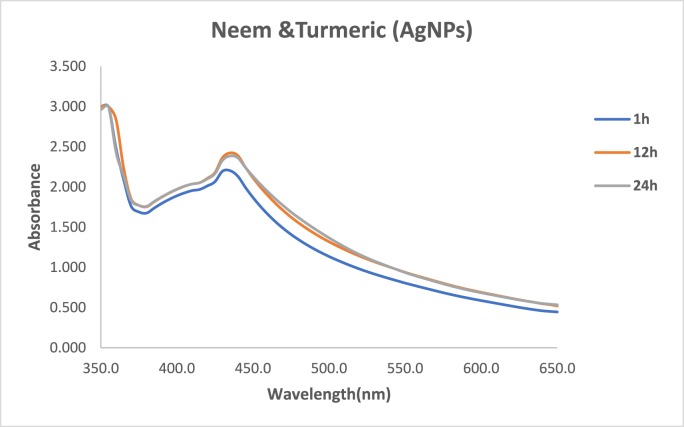
UV–Visible spectra of green synthesized silver nanoparticles. (For interpretation of the references to color in this figure legend, the reader is referred to the Web version of this article.)

**Graph 2:**
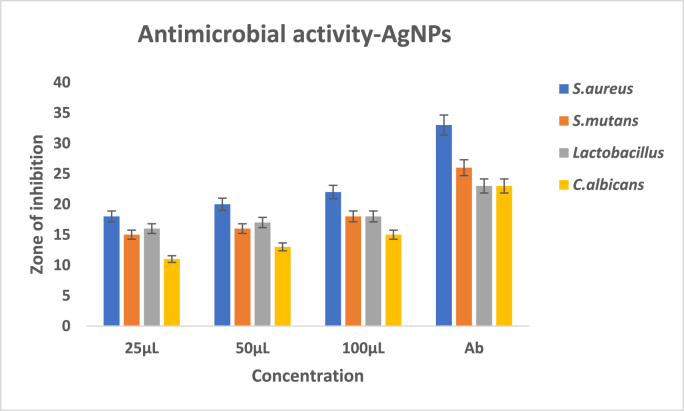
Antimicrobial activity of silver nanoparticles against oral pathogens.

The increasing concerns surrounding the overuse of antibiotics and the rapid proliferation of drug-resistant microbial strains have sparked a quest for alternative antimicrobial solutions. A promising avenue of exploration lies in the utilization of metallic nanoparticles, with a particular focus on silver nanoparticles, for their robust antimicrobial properties.^1^^,^^2^ (see Fig. 5, Graph 1, Graph 2)

Silver nanoparticles (AgNPs) are renowned for their wide-ranging antimicrobial capabilities and have found applications in diverse fields, including wound care, medical device coatings, and water purification systems. However, conventional methods for synthesizing AgNPs frequently entail the use of hazardous chemicals and result in the generation of toxic by-products.^3^^,^^4^

In response to these challenges, there has been a growing interest in the green synthesis of silver nanoparticles. This eco-friendly approach leverages biological entities such as plant extracts, bacteria, fungi, or enzymes as both reducing and capping agents during nanoparticle synthesis. This not only mitigates environmental harm but also enhances biocompatibility, rendering the nanoparticles more suitable for biomedical purposes.^5^, ^6^, ^7^

Among the array of biological entities employed in green synthesis, plant extracts stand out as a highly promising option due to their widespread availability, safety profile, and the presence of a diverse array of bioactive compounds that aid in nanoparticle synthesis. Notably, extracts derived from neem and turmeric have demonstrated effectiveness in silver nanoparticles (AgNPs)synthesis. Both neem and turmeric have long been recognized for their medicinal properties and have played pivotal roles in traditional medicine for centuries.^8^^,^^9^ The bioactive compounds within these extracts not only facilitate AgNP synthesis but also augment their antimicrobial potency.

This research endeavors to delve into the green synthesis of silver nanoparticles using neem and turmeric extracts, further probing their antimicrobial efficacy. The outcomes of this study may contribute significantly to the development of novel, environmentally friendly antimicrobial agents, poised to combat the pressing issue of antibiotic resistance.

### 1.1 Unique aspects of the study

• Combines neem and turmeric extracts, unlike previous studies that focus on single plant sources, to potentially enhance nanoparticle potency.
• Targets specific oral pathogens (*Candida albicans, Lactobacillus, Staphylococcus aureus,* and *Streptococcus mutans*), making it highly relevant for oral healthcare.
• Employs rigorous characterization techniques (UV–Vis, SEM, EDX, XRD) to ensure precise analysis of nanoparticle properties.
• Evaluates the dose-dependent antimicrobial effects of the synthesized AgNPs, offering insights into optimized therapeutic use.

### 1.2 Rationale of the study

This study addresses the need for alternative antimicrobial solutions due to increasing antibiotic resistance and the environmental hazards of conventional silver nanoparticle (AgNP) synthesis. By leveraging the medicinal properties of neem and turmeric, the research explores an eco-friendly green synthesis method for AgNPs that enhances biocompatibility and antimicrobial efficacy.

## 2 Materials and Methods

### 2.1 Preparation of Neem and turmeric aqueous formulation

An aqueous formulation was prepared by combining 1g of Neem leaves powder and 1g of turmeric powder in 100 mL of distilled water. The mixture was gently stirred to ensure even dispersion, and then heated using a heating mantle at 60 °C for 15–20 min. This heating process aimed to facilitate the extraction of active components from the powders and promote dissolution into the water. After heating, the mixture was filtered through Whatman No:1 filter paper to separate the aqueous solution from the solid residues of Neem leaves and turmeric powder. The resulting filtrate was collected and stored in a clean container.

### 2.2 Green synthesis of silver nanoparticles

A 1 ml solution of silver nitrate was employed as the precursor for synthesizing silver nanoparticles, which were then dispersed in 80 mL of distilled water. Following this, a mixture of 20 mL of neem and turmeric filtered extracts was mixed into the solution. The resultant mixture was subjected to constant stirring at 700 rpm using a magnetic stirrer for uniform dispersion over a period of 24–48 h. Subsequently, the solution underwent centrifugation to separate the pellet from the supernatant. The pellet was carefully collected and subjected to drying in a hot air oven at 80 °C to obtain a powdered form suitable for characterization, while the supernatant was discarded.

### 2.3 Characterization

To characterize the silver nanoparticles synthesized using neem and turmeric, UV–Visible spectrophotometry (Make: Shimadzu, Model: UV-1800, Japan) was employed to assess their optical properties and Scanning Electron microscope (Make: JEOL, Model: JSM-7610F, Japan) for the determining the shape and Elemental Dispersive X-Ray Analysis to confirm the presence of elements in the synthesized solution. Additionally, X-ray diffraction (XRD Make: Bruker, Model: D8 Advance, Germany) analysis was conducted to determine the crystalline size of the nanoparticles.

### 2.4 Antimicrobial activity

The antimicrobial properties of green synthesized silver nanoparticles were evaluated in this study using the agar well diffusion technique. Four oral pathogens, namely *Candida albicans, Lactobacillus, Staphylococcus aureus, and Streptococcus mutans,* were selected as test organisms obtained from a pathogen bank maintained at the laboratory. Mueller-Hinton Agar was employed as the culture medium to determine the zones of inhibition. The preparation and sterilization of Mueller-Hinton Agar plates were conducted at 121 °C for 15 min. Subsequently, the agar was poured into sterilized plates and allowed to solidify. Wells with a diameter of 9 mm were created using sterile polystyrene tips, and the test organisms were swabbed onto the agar plates using sterile cotton swabs. To assess antimicrobial activity, neem and turmeric-mediated silver nanoparticles were loaded into three separate wells with different volume (25 μL, 50 μL, and 100 μL), while the fourth well served as a positive control containing Amoxyrite (Amoxicillin (10 μg/disc). Amoxyrite was selected as the control antibiotic due to its broad-spectrum. *Amoxicillin* (10 μg/disc) was used as the positive control to validate the assay and serve as a benchmark for comparing the efficacy of the synthesized silver nanoparticles. The bacterial plates were then incubated at 37 °C for 24 h, after which the zones of inhibition were measured.

### 2.5 Growth, maintenance, and identification of pathogen cultures

The oral pathogens used in this study, namely *Candida albicans*, *Lactobacillus* spp., *Staphylococcus aureus*, and *Streptococcus mutans*, were procured from pathogen bank. The pathogens were cultured on their respective recommended growth media to ensure viability and purity. Bacterial strains were maintained on Mueller-Hinton Agar (MHA) plates and subcultured at 37 °C for 24 h before use. Fungal cultures were maintained on Sabouraud Dextrose Agar (SDA) under similar conditions.

Identification of the strains was confirmed via Gram staining, to rule out contamination from environmental pathogens. All assays were conducted under aseptic conditions to prevent contamination.

### 2.6 Preparation of bacterial suspension

The bacterial suspension was standardized to ensure uniformity in the number of bacteria plated. Pathogen cultures were adjusted to match a 0.5 McFarland standard, equivalent to approximately 1.5 × 10^8^ CFU/mL using a spectrophotometer at 600 nm. Following standardization, 100 μL of the bacterial suspension was evenly spread onto the agar plates using a sterile swab.

## 3 Result and discussion

### 3.1 Visual observation

**Figure:1:**
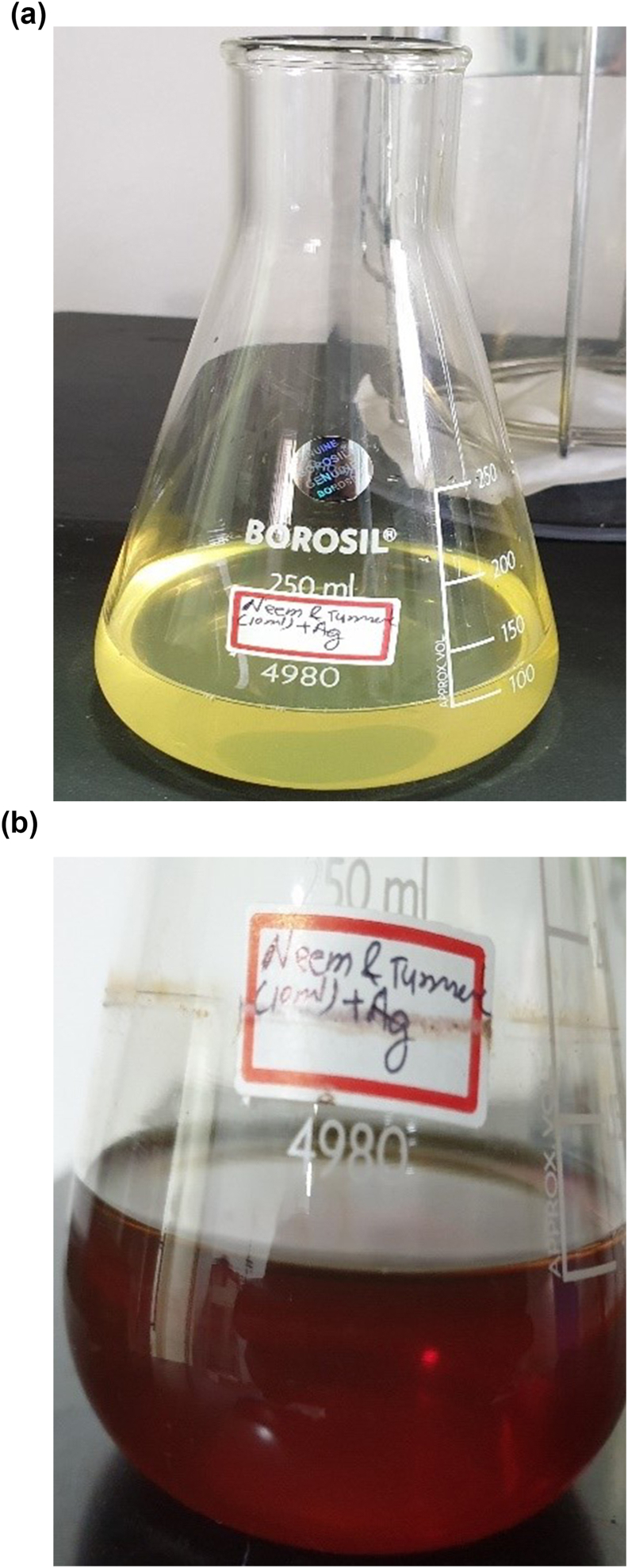
a & b.

In this study, we investigated the green synthesis of silver nanoparticles (AgNPs) using neem and turmeric extract as reducing and capping agents. Visual observations of the initial and final images of the synthesized AgNPs were depicted in Fig. 1.

Upon the addition of neem and turmeric extract to the silver nanoparticle synthesis solution, a distinct color change was observed. The initial image exhibited a vibrant golden-yellow hue (Fig. 1a), indicating the formation of AgNPs. This color change is a characteristic feature of silver nanoparticles formation in the presence of biogenic reducing agents such as neem and turmeric. After allowing the reaction to proceed for 48 h, the color of the synthesized AgNPs solution had evolved to a distinct reddish-brown hue (Fig. 1b). This transformation in color is indicative of the growth and stabilization of AgNPs. The change in color from golden-yellow to reddish-brown suggests a modification in the size and shape of the nanoparticles over time.^10^^,^^11^

The observed color changes in the initial and final images provide visual evidence of the successful synthesis of silver nanoparticles using neem and turmeric extract as green reducing and capping agents. The shift in color from golden-yellow to reddish-brown signifies the reduction of silver ions and the formation of stable AgNPs.

### 3.2 UV–visible spectroscopy

**Fig. 2:**
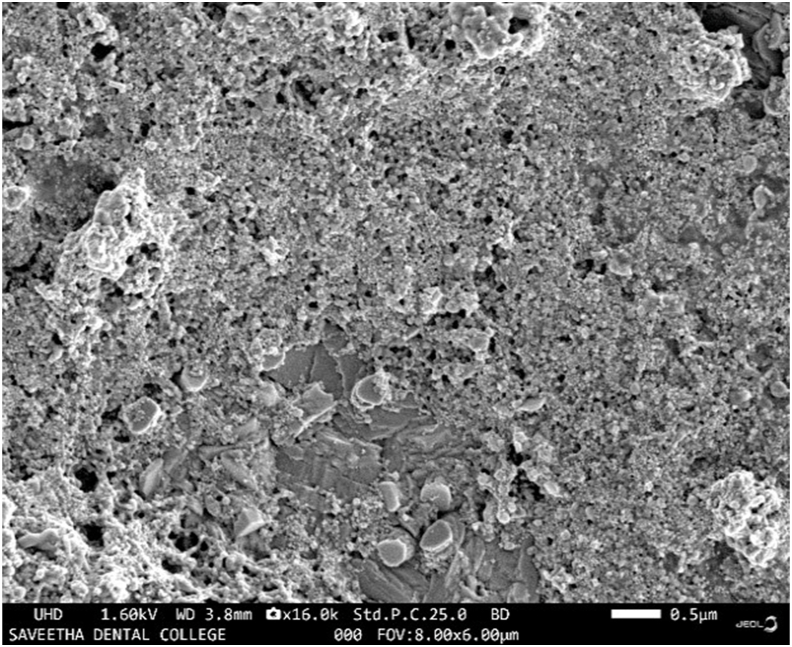
SEM image of neem and turmeric mediated silver nanoparticles.

The UV–Visible spectrum of the green synthesized silver nanoparticles (AgNPs) was obtained to provide further insight into their optical properties. Fig. 2 depicts the UV–Visible spectrum, where a maximum absorption peak was observed at 440 nm.The absorption peak at 440 nm is a characteristic feature of silver nanoparticles and is associated with their surface plasmon resonance (SPR). This phenomenon occurs due to the collective oscillation of conduction band electrons in the metal nanoparticles when subjected to incident light.^12^^,^^13^ The position and intensity of the SPR peak are influenced by the size, shape, and aggregation state of the nanoparticles.

In this study, the observed maximum absorption peak at 440 nm confirms the presence of silver nanoparticles in the synthesized solution. The position of the peak aligns with the typical SPR wavelength for silver nanoparticles, indicating the successful formation of nanoparticles with the green synthesis approach using neem and turmeric extract as reducing and capping agents.^14^

### 3.3 Scanning electron microscopy (SEM)

**Fig. 3:**
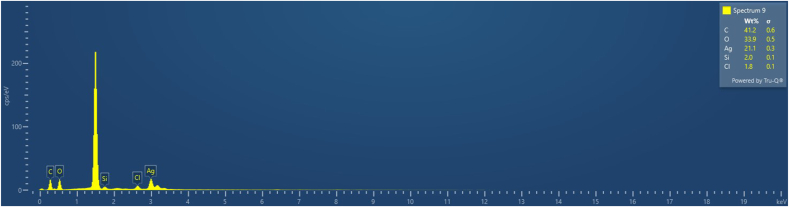
EDAX image of neem and turmeric mediated silver nanoparticles.

The scanning electron microscope (SEM) image of neem and turmeric-mediated silver nanoparticles (AgNPs) reveals distinctive structural characteristics which was depicted in Fig. 3. The green-synthesized AgNPs exhibit a small and spherical morphology. This SEM image provides visual evidence of the nanoparticle size, shape, and uniformity, confirming their spherical nature.

The spherical shape of these AgNPs is a noteworthy attribute, as it can impact their properties and potential applications. Spherical nanoparticles often have a high surface area-to-volume ratio, which can be advantageous for various applications in fields such as catalysis, electronics, and medicine. Additionally, the size and shape of nanoparticles can influence their optical and antimicrobial properties, making them a subject of interest in diverse scientific research areas.^15^^,^^16^**.**

### 3.4 Energy dispersive X-Ray Analysis

**Fig. 4:**
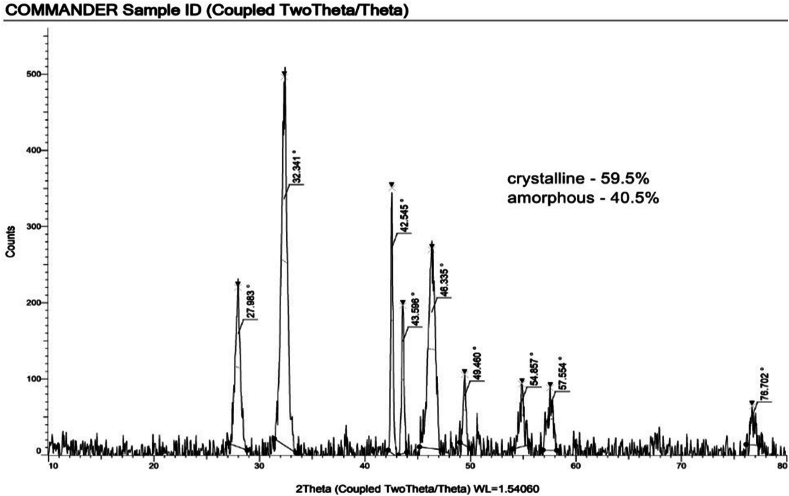
XRD spectra of green synthesized silver nanoparticles.

The Energy Dispersive X-ray Analysis (EDAX) was employed to determine the elemental composition of neem and turmeric-mediated silver nanoparticles (AgNPs) which was depicted in Fig. 4 The analysis revealed the presence of several elements within the sample, and the relative composition by weight percentage is as follows:Carbon (C): 41.2 %, Oxygen (O): 33.9 %, Silver (Ag): 21.1 %, Silicon (Si): 2.0 %, Chlorine (Cl): 1.8 %

**Fig. 5:**
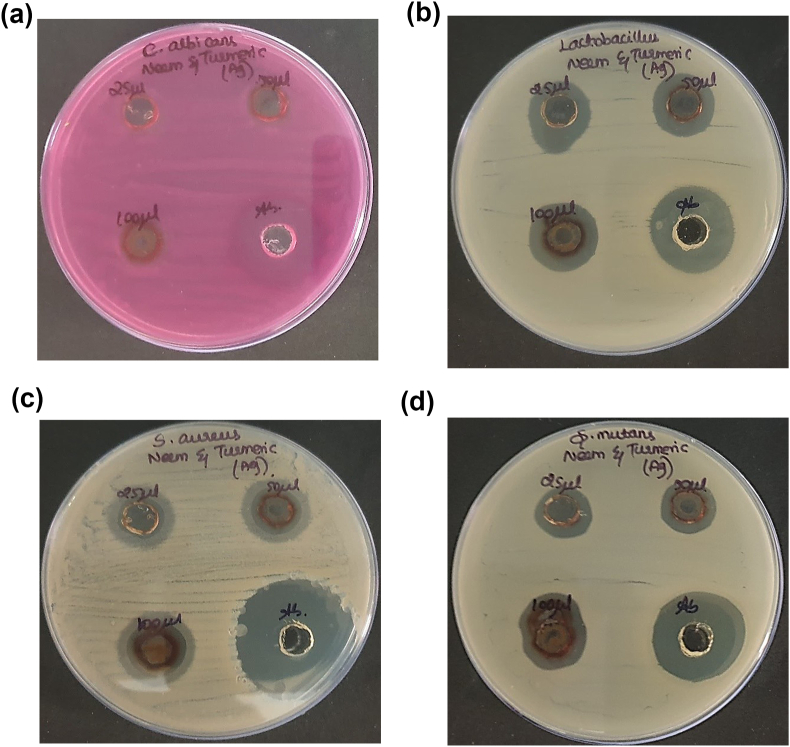
Antimicrobial activity of neem and turmeric mediated silver nanoparticles using agar well diffusion technique. a) *Candida albicans* b) *Lactobacillus* c) *Staphylococcus aureus* d) *Streptococcus mutans*.

These results indicate the predominant elemental constituents of the synthesized nanoparticles. Notably, silver (Ag) is a significant component, aligning with the intended synthesis of silver nanoparticles using neem and turmeric extracts. The presence of carbon (C) and oxygen (O) suggests the involvement of organic components from the plant extracts in stabilizing and reducing the silver ions during the green synthesis process. The minor presence of silicon (Si) and chlorine (Cl) could be attributed to trace impurities or interactions with the surrounding environment during sample preparation and analysis.

A study conducted by Rajkuberan et al. demonstrated the remarkable antibacterial activity of silver nanoparticles that were synthesized utilizing neem extract. The EDAX analysis in this study served to validate the presence of silver nanoparticles.^17^

Overall, the EDAX analysis provides valuable insights into the elemental composition of the neem and turmeric-mediated silver nanoparticles, confirming the successful synthesis of silver nanoparticles with a predominant silver content along with organic components.

### 3.5 X-ray diffraction analysis

The X-ray diffraction (XRD) analysis of green-synthesized silver nanoparticles reveals essential structural characteristics. The data indicates that 59.5 % of the sample comprises a crystalline phase, while the remaining 40.5 % exists in an amorphous state. This observation suggests the presence of well-defined crystal structures within a substantial portion of the silver nanoparticles.

To estimate the average crystallite size of the silver nanoparticles, we employed the Debye Scherrer equation.

D = 0.9λ / βcos(θ)

Where,

D represents the average crystallite size.

λ is the X-ray wavelength (usually taken as 1.5406 Å for Cu Kα radiation).

β is the full width at half maximum (FWHM) of the XRD peak.

θ is the Bragg angle corresponding to the peak.

For Peak 1 at 2θ = 27.983° with a FWHM of 0.329°, the estimated size (D1) is approximately 6.63 nm. For Peak 2 at 2θ = 32.341° with a FWHM of 0.372°, the estimated size (D2) is approximately 5.74 nm. For Peak 3 at 2θ = 42.545° with a FWHM of 0.133°, the estimated size (D3) is approximately 14.81 nm. For Peak 4 at 2θ = 43.596° with a FWHM of 0.158°, the estimated size (D4) is approximately 12.55 nm. For Peak 5 at 2θ = 46.335° with a FWHM of 0.463°, the estimated size (D5) is approximately 4.29 nm. For Peak 6 at 2θ = 49.460° with a FWHM of 0.299°, the estimated size (D6) is approximately 7.00 nm. For Peak 7 at 2θ = 54.857° with a FWHM of 0.536°, the estimated size (D7) is approximately 3.79 nm. For Peak 8 at 2θ = 57.554° with a FWHM of 0.605°, the estimated size (D8) is approximately 3.39 nm. For Peak 9 at 2θ = 76.702° with a FWHM of 0.523°, the estimated size (D9) is approximately 4.14 nm.

The green synthesis of silver nanoparticles (AgNPs) and their subsequent characterization using XRD spectroscopy have been the focus of extensive research in recent studies. Various plant extracts have been employed for the eco-friendly synthesis of AgNPs, with researchers investigating their size, structure, and potential applications.

X-ray diffraction (XRD) spectroscopy serves as a valuable tool for the precise determination of the size and crystal structure of nanoparticles. A recent study by Khalil et al., in 2023 exemplifies the use of Piper cubeba extract as a reducing agent for the green synthesis of AgNPs. In this research, the XRD patterns obtained for the synthesized AgNPs, prepared using the *P. cubeba* extract, exhibited four distinct diffraction peaks. These findings unequivocally confirmed the crystalline nature and face-centered-cubic (FCC) crystal structure of the produced AgNPs. Moreover, the study's analysis revealed that the mean crystalline diameters of the AgNPs were measured to be 7, 15, 38, and 51 nm, respectively.^18^

The size distribution of green-synthesized silver nanoparticles, ranging from 4 nm to 14.81 nm, signifies that these nanoparticles exhibit a range of sizes within this specific sample. Therefore, the observed size distribution provides valuable information for understanding the structural characteristics of the green-synthesized silver nanoparticles and their potential utility in various applications, depending on the desired size range and properties.

### 3.6 Antimicrobial activity

The antimicrobial activity of silver nanoparticles synthesized using neem and turmeric was evaluated in comparison to the standard antibiotic, amoxyrite, through the measurement of the zone of inhibition. For *Staphylococcus aureus* (S. aureus), the silver nanoparticles displayed zones of inhibition of 18 mm (25 μL), 20 mm (50 μL), and 20 mm (100 μL), while amoxyrite exhibited a 33 mm zone of inhibition. In the case of *Streptococcus mutans* (*S. mutans*), the silver nanoparticles exhibited zones of inhibition measuring 15 mm (25 μL), 16 mm (50 μL), and 16 mm (100 μL), while amoxyrite showed a 26 mm zone of inhibition. Against Lactobacillus sps, the silver nanoparticles displayed zones of inhibition measuring 16 mm (25 μL), 17 mm (50 μL), and 18 mm (100 μL), whereas amoxyrite had a 23 mm zone of inhibition. Lastly, for *Candida albicans* (C. albicans), the silver nanoparticles exhibited zones of inhibition of 11 mm (25 μL), 13 mm (50 μL), and 13 mm (100 μL), while amoxyrite had a 23 mm zone of inhibition. These results indicate the potential of neem and turmeric-mediated silver nanoparticles as antimicrobial agents, especially against *S. aureus*, *S. mutans*, and Lactobacillus sps.

The eco-friendly and cost-effective approach of green synthesis of silver nanoparticles using natural plant extracts has garnered significant interest in recent years. This method utilizes extracts from plants and microorganisms to act as both reducing and capping agents during the production of silver nanoparticles, hence termed “green synthesis.” Numerous research studies have demonstrated the potent antimicrobial properties of green-synthesized silver nanoparticles against a range of oral pathogens, including Streptococcus mutans, *Staphylococcus aureus*, and Candida.

In previous research work, anticariogenic activity of nano silver fluoride was studied and the findings revealed that nano silver fluoride exhibits the ability to remineralize enamel, particularly in deciduous teeth, even at lower concentrations. Furthermore, it demonstrates bactericidal properties, establishing its potential as an effective anticariogenic agent.^19^

Another investigation into the biomedical applications of plant extract mediated silver nanoparticles revealed that green synthesized silver nanoparticles possess antimicrobial activities that are comparable to, and in some cases superior to, conventional antimicrobial agents. Furthermore, when combined with traditional drugs, these green-synthesized silver nanoparticles display synergistic effects, enhancing their overall antimicrobial activity.^20^

Expanding on the applications of silver nanoparticles in dentistry, Fernandes et al., 2021 reported about their versatility in disinfection, prophylaxis, and the prevention of infections within the oral cavity. Their potential in caries prevention and treatment is emphasized, highlighting their significant role in oral health.^21^Additionally, Sneha et al., 2017 demonstrated that silver nanoparticles synthesized with turmeric extract possess robust antimicrobial activity against specific microorganisms, including *Bacillus subtilis* and *Pseudomonas aeruginosa*.^13^^,^^22^

Overall, these studies collectively underscore the valuable role of silver nanoparticles in dentistry, where they exhibit substantial antimicrobial activity against various microorganisms and hold great promise for oral health applications.

## 4 Conclusion

The conducted research has successfully showcased the potential of neem and turmeric extracts in the green synthesis of silver nanoparticles (AgNPs). These plant-mediated AgNPs have demonstrated remarkable antimicrobial activity against a range of oral pathogens, thereby confirming their viability as an alternative to conventional antibiotics.The green synthesis approach not only adheres to the principles of sustainability and eco-friendly chemistry but also offers a straightforward, cost-effective, and environmentally conscious method for AgNP synthesis. Moreover, the utilization of neem and turmeric extracts, renowned for their medicinal attributes, has enriched the synthesized AgNPs by amplifying their antimicrobial effectiveness. Beyond their antimicrobial attributes, the synthesized AgNPs have displayed commendable stability, rendering them suitable for diverse biomedical applications. The outcomes of this research carry significant implications for the advancement of novel antimicrobial agents and contribute valuably to the expanding realm of knowledge concerning green nanoparticle synthesis.

## Patients consent

Patient consent is not required.

## Ethics

Since this is an original laboratory study, no ethical clearance was taken from any institution.

## Source of funding

Self-funded study.

## Declaration of competing interest

The authors declare that they have no known competing financial interests or personal relationships that could have appeared to influence the work reported in this paper.
